# A Hydrocarbon Soluble, Molecular and “Complete” Al-Cocatalyst for High Temperature Olefin Polymerization

**DOI:** 10.3390/polym15061378

**Published:** 2023-03-09

**Authors:** Gaia Urciuoli, Francesco Zaccaria, Cristiano Zuccaccia, Roberta Cipullo, Peter H. M. Budzelaar, Antonio Vittoria, Christian Ehm, Alceo Macchioni, Vincenzo Busico

**Affiliations:** 1 Department of Chemical Sciences, Federico II University of Naples, 80126 Napoli, Italy; 2 Department of Chemistry, Biology and Biotechnology and CIRCC, University of Perugia, 06123 Perugia, Italy; 3 Dutch Polymer Institute (DPI), P.O. Box 902, 5600 AX Eindhoven, The Netherlands

**Keywords:** olefin polymerization, catalyst activation, borate activators, methylaluminoxane, soluble cocatalyst

## Abstract

The dinuclear aluminum salt {[*i*Bu_2_(DMA)Al]_2_(*μ*-H)}^+^[B(C_6_F_5_)_4_]^−^ (**AlHAl**; DMA = *N*,*N*-dimethylaniline) is the prototype of a new class of molecular cocatalysts for catalytic olefin polymerization, its modular nature offering easy avenues for tailoring the activator to specific needs. We report here, as proof of concept, a first variant (**s-AlHAl**) bearing *p*-hexadecyl-*N*,*N*-dimethylaniline (DMA_C16_) units, which enhances solubility in aliphatic hydrocarbons. The novel **s-AlHAl** was used successfully as an activator/scavenger in ethylene/1-hexene copolymerization in a high-temperature solution process.

## 1. Introduction

Several high-performance polyolefin resins are produced with molecular catalysts in solution [1,2,3]. The active species are typically ion pairs of a cationic group 4 metal-alkyl complex and a weakly coordinating anion [4]. Said ion pairs are generated in situ by the reaction between a neutral precatalyst and a cocatalyst (activator), both playing key roles; in fact, the success story of industrial molecular olefin polymerization catalysis must be traced to the identification of high-performing combinations of (pre)catalysts [2,3,4,5,6,7,8] and cocatalysts [9,10,11,12,13]. This notwithstanding, precatalyst diversification attracted great attention, whereas reports on cocatalysts are more limited [14,15,16,17,18,19,20,21,22,23,24,25].

**Figure 1:**
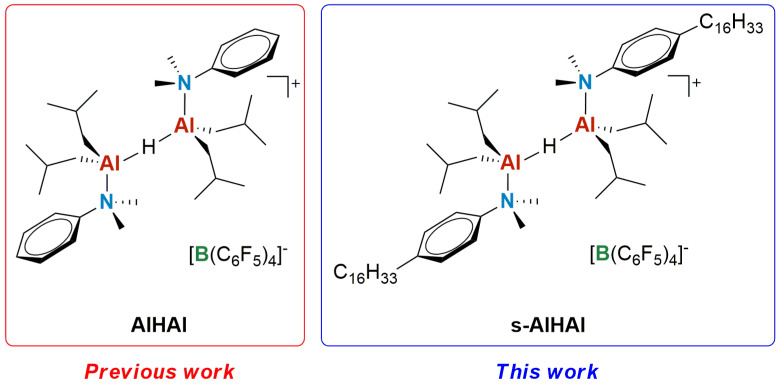
Structures of **AlHAl** and its hydrocarbon soluble analogue **s-AlHAl**.

In the framework of a more general study of the aforementioned activation process [26], we have recently identified a novel Al-based cocatalyst, namely {[*i*Bu_2_(PhNMe_2_)Al]_2_(*μ*-H)}^+^[B(C_6_F_5_)_4_]^−^ (**AlHAl**, Figure 1, left) [27]. This unusual homodinuclear cation has a distinctive bridging hydride between two Al atoms. While the two Al centers are coordinatively saturated, **AlHAl** possesses “latent” Lewis acidity; indeed, we reported indications that it can release the unsaturated [Al*i*Bu_2_(PhNMe_2_)]^+^ fragment [27], resembling [AlMe_2_]^+^ release from methylalumoxane (MAO) [28,29,30,31,32,33]. **AlHAl** can be viewed as a “tamed” version of the highly unstable [Al*i*Bu_2_]^+^ species [34,35], and yet it is able to competently activate group 4 metal precatalysts (and on top of that to scavenge impurities). Activation of a prototypical *ansa*-zirconocene precatalyst turned out to occur at [Al]/[Zr] and [Zr] values as low as 50 and ~10^−7^ M, respectively [27]; catalyst performance in propene polymerization was comparable to that with MAO activation, even though the latter required 100-fold to 1000-fold larger [Al]/[Zr]. Moreover and at odds with other well-known molecular activators (e.g., borate salts of organic Lewis and Brønsted acids) [4,10], **AlHAl** is able to activate dichloride precatalysts with no need for a separate alkylating and scavenging agent, like e.g., tri-*iso*-butylaluminum (TIBAL). Last but not least, the ease of synthesis and the modular nature of **AlHAl** (comprised of Al-alkyl groups, neutral N-ligands, a bridging hydride and a borate anion) makes it the prototype of a new cocatalyst class, potentially opening the door to tailored applications.

On the other hand, like all ionic activators (and MAO too) **AlHAl** is poorly soluble in aliphatic hydrocarbons, which represents a major drawback for use in homogeneous phase [16,19,20,24,36,37,38]. To overcome this problem, we modified the N-donor fragments of **AlHAl** by installing *p*-hexadecyl residues on the phenyl rings of aniline. In this paper, we report on the synthesis and characterization of the thus obtained **s-AlHAl** species (Figure 1, right), its solubility in pentane, and the performance in the activation of a representative *ansa*-zirconocene. The productivity of the resulting catalyst system in hydrocarbon solution at high temperature was evaluated in ethene/1-hexene copolymerization, comparatively with conventional activators.

## 2. Materials and Methods

All manipulations were performed under rigorous exclusion of oxygen and moisture in flame-dried Schlenk-type glassware interfaced to a high-vacuum line (<10^−5^ Torr), or in a nitrogen-filled MBraun glovebox (<0.5 ppm O_2_). Molecular sieves (4Å, MS) were activated for 24 h at ca. 200–230 °C under dynamic vacuum. Benzene-*d*_6_ and toluene-*d*_8_ were freeze–pump–thaw degassed on a high-vacuum line, dried over Na/K alloy, vacuum-transferred to a dry storage tube with a PTFE valve and stored over activated MS in the glovebox. TTB (Boulder Scientific Co., Longmont, Colorado), AB (Boulder Scientific Co., Longmont, CO, USA), TIBAL (Merck, Milan, Italy), DIBAL-H (Merck, Milan, Italy), 2M HCl in ether (Merck, Milan, Italy), 2,6-di-tert-butyl-4-methylphenol (BHT; Merck, Milan, Italy), Li[B(C_6_F_5_)_4_] etherate complex (abcr), DMA_C16_ (Ricci Chimica, Perugia, Italy), and 1,2-dichlorobenzene (>99.8% isomeric purity; Romil, Cambridge, UK) were purchased and used as received. Ethene (99.95%; Linde, Arluno—MI, Italy) was purified by flowing through a column containing activated 4 Å molecular sieves and an activated Cu catalyst (BASF R0-11G). 1-hexene (99%; Merck, Milan, Italy) was purified by passing it through a mixed-bed column of activated Cu catalyst and 4 Å molecular sieves. Toluene (Romil, Cambridge, UK), pentane (Romil, Cambridge, UK), and ISOPAR-G were dried by using an MBraun SPS-5 solvent purification unit. Dichloromethane (Carlo Erba, Cornaredo, Italy) was dried over calcium hydride, distilled and freeze–pump–thaw degassed on a high-vacuum line. **Cat-Zr** was synthesized according to reported procedures [39] and kindly donated by the group of Prof. A. Voskoboynikov from Lomonosov Moscow State University, Russia.

### 2.1. NMR Spectroscopy Experiments

NMR experiments were performed by using a Bruker Avance III HD 400 instrument equipped with a smart probe (400 MHz for ^1^H). ^1^H NMR spectra were referenced to the residual protons of the deuterated solvent used; ^13^C NMR spectra were referenced internally to the D-coupled ^13^C resonances of the NMR solvent. To describe the multiplicity of the signals, the following abbreviations are used: s, singlet; bs, broad singlet; d, doublet; bd, broad doublet; dd, doublet of doublets; t, triplet; and m, multiplet.

DOSY NMR measurements were performed at 298 K in pentane by using a coaxial capillary containing acetone-*d*_6_. The standard double-stimulated echo pulse sequence without spinning was used. The shape of the gradients was rectangular, their duration (d) was 4 ms, and their strength (G) was varied during the experiments. All the spectra were acquired by using 16 K data points, 32 increments, between 16 and 128 scans, a spectral width of 6000 Hz, an acquisition time of 1.3 s, and a relaxation delay of 2 s per transient, and processed by using the inverse Laplace transform (ILT) routine of the Bruker Dynamics Center software suite. Pentane was used as internal standard to account for temperature and viscosity fluctuations; its *D*_t_ was calibrated by using an external sample of HDO in D_2_O under the same conditions [40]. The *D*_t_ data were treated as described in the literature to derive the hydrodynamic dimensions [41,42]. The uncertainties were estimated by assuming an error of approximately 5% in the hydrodynamic radius, and 15% in hydrodynamic volume and aggregation number [43,44]; error intervals in *K*_aggr_ are given at 95% confidence.

The average aggregation number was calculated as the ratio between the average hydrodynamic volume of the aggregate (*V*_H_) and that of the single ion pair (*V*_H_^0^) [45]. The latter was estimated as the sum of the hydrodynamic volume of the cation (*V*_H_^+0^) and the anion (*V*_H_^−0^) by assuming that

*V*_H_^0^ = *V*_H_^+0^ + *V*_H_^−0^ = 2 × (*V*_H_^DMAC16^ + 2/3 × *V*_H_^TIBAL^) + *V*_H_^−0^,

where the hydrodynamic volume of the free **DMA_C16_** molecule (*V*_H_^DMAC16^ = 659 ± 99 Å^3^) and that of TIBAL (*V*_H_^TIBAL^ = 381 ± 57 Å^3^) were estimated by performing ad-hoc DOSY NMR experiments, whereas that of the borate anion (*V*_H_^−0^ = 605 ± 91 Å^3^) was obtained from the literature [44], resulting in *V*_H_^0^ = 2432 ± 365 Å^3^. 

Experimental trends of *N* vs. *C* (see Section 3.1) were analyzed as reported in the literature [44] by using three different models for indefinite self-aggregation data: EK (equal K), IK (incremental K), and AK (attenuated K) [46]. The IK gave a better fit than the EK model (as determined by the Euclidean norm of the residuals with respect to the experimental *N* values, being 0.594 and 9.510, respectively), whereas the AK model was unable to reach convergence.

### 2.2. Synthetic Procedures

**[(*p*-C_16_H_33_)PhN(Me)_2_H]^+^[B(C_6_F_5_)_4_]**^−^. Taking inspiration from literature procedures [47], a 2 M solution of HCl in diethyl ether (0.44 mL, 0.88 mmol) was added dropwise to a stirred solution of DMA_C16_ (304 mg, 0.88 mmol) in pentane (15 mL). A fluffy white solid rapidly precipitated, and the resulting suspension was stirred at room temperature for 30 min. The volatiles were removed under vacuum. The white residue, corresponding to [(*p*-C_16_H_33_)PhN(Me)_2_H]Cl, was suspended in fresh pentane and isolated by filtration (275 mg). An aliquot of [(*p*-C_16_H_33_)PhN(Me)_2_H]Cl (150 mg, 0.39 mmol) was then transferred in a Schlenk flask along with Li[B(C_6_F_5_)_4_] etherate complex (352 mg, 0.39 mmol) and dissolved in dichloromethane (10 mL). The resulting turbid mixture was stirred at room temperature for 3 h, observing formation of a white precipitate over time. The volatiles were removed under vacuum and the residue was extracted with toluene, filtered, dried under vacuum, and washed with pentane to obtain [(*p*-C_16_H_33_)PhN(Me)_2_H]^+^[B(C_6_F_5_)_4_]^−^ as a white solid (277 mg, 66% yield).

^1^H NMR (400 MHz, benzene-*d_6_*, 298 K): 6.83 (d, 2H, ^3^*J* = 8.8 Hz, aromatic *m*-CH), 6.15 (d, 2H, aromatic *o*-CH), 5.13 (bs, 1H, N-H), 2.34 (t, 2H, ^3^*J* = 7.6 Hz, -C*H_2_*-CH_2_-(CH_2_)_12_-CH_2_-CH_3_), 1.66 (s, 6H, N-CH_3_), 1.43 (m, 2H, -CH_2_-C*H_2_*-(CH_2_)_12_-CH_2_-CH_3_), 1.37–1.27 (m, 24H, -CH_2_-CH_2_-(C*H_2_*)_12_-CH_2_-CH_3_), 1.30 (m, 2H, -CH_2_-CH_2_-(CH_2_)_12_-C*H_2_*-CH_3_), 0.91 (t, 3H, ^3^*J* = 7.0 Hz, -CH_2_-CH_2_-(CH_2_)_12_-CH_2_-C*H_3_*) ppm. ^13^C NMR (100 MHz, benzene-*d_6_*, 298 K): 147.3 (quaternary aromatic C), 137.1 (quaternary aromatic C-N), 130.5 (aromatic *m*-CH), 118.1 (aromatic *o*-CH), 46.5 (N-CH_3_), 35.0 (-*C*H_2_-CH_2_-(CH_2_)_12_-CH_2_-CH_3_), 31.0 (-CH_2_-*C*H_2_-(CH_2_)_12_-CH_2_-CH_3_), 29.9–29.1 (-CH_2_-CH_2_-(*C*H_2_)_12_-CH_2_-CH_3_), 22.7 (-CH_2_-CH_2_-(CH_2_)_12_-*C*H_2_-CH_3_), 14.0 (-CH_2_-CH_2_-(CH_2_)_12_-CH_2_-*C*H_3_) ppm.

**s-Al and s-AlHAl.** TIBAL (42 µL, 166 mmol) was added to a stirred suspension of [(*p*-C_16_H_33_)PhN(Me)_2_H]^+^[B(C_6_F_5_)_4_]^−^ (150 mg, 141 mmol) and DMA_C16_ (54 mg, 156 mmol) in toluene (2 mL) at room temperature. The mixture rapidly turns from deep to pale orange, and effervescence is observed. After stirring for 16h, the volatiles were removed under vacuum, and the residue washed with pentane to give **s-Al** as a pale orange ionic liquid (170 mg, 80% yield). An aliquot of **s-Al** (30 mg, 20 mmol) was then dissolved in a solution of DIBAL-H (20 mmol) in ISOPAR-G (8 mL, for polymerization experiments) or pentane (8 mL, for synthetic purposes) to obtain **s-AlHAl**, which can be isolated in quantitative yield as a pale orange oil upon removal of volatiles under vacuum.

**s-Al**. ^1^H NMR (400 MHz, benzene-*d_6_*, 298 K): 7.00 (d, 4H, ^3^*J* = 8.8 Hz, aromatic *m*-CH), 6.65 (d, 4H, aromatic *o*-CH), 2.50 (t, 4H, ^3^*J* = 7.8 Hz, -C*H_2_*-CH_2_-(CH_2_)_12_-CH_2_-CH_3_), 1.99 (s, 12H, N-CH_3_), 1.55 (m, 4H, -CH_2_-C*H_2_*-(CH_2_)_12_-CH_2_-CH_3_), 1.51 (m, 2H, Al-CH_2_-C*H*(CH_3_)_2_), 1.40–1.29 (m, 48H, -CH_2_-CH_2_-(C*H_2_*)_12_-CH_2_-CH_3_), 1.30 (m, 4H, -CH_2_-CH_2_-(CH_2_)_12_-C*H_2_*-CH_3_), 0.95 (d, 12H, ^3^*J* = 6.4 Hz, Al-CH_2_-CH(C*H_3_*)_2_), 0.92 (t, 6H, ^3^*J* = 7.0 Hz, -CH_2_-CH_2_-(CH_2_)_12_-CH_2_-C*H_3_*), 0.05 (d, 4H, ^3^*J* = 6.8 Hz, Al-C*H*_2_-CH(CH_3_)_2_) ppm. ^13^C NMR (100 MHz, benzene-*d_6_*, 298 K): 144.1 (quaternary aromatic C), 142.4 (quaternary aromatic C-N), 129.7 (aromatic *m*-CH), 120.4 (aromatic *o*-CH), 46.7 (N-CH_3_), 34.8 (-*C*H_2_-CH_2_-(CH_2_)_12_-CH_2_-CH_3_), 31.2 (-CH_2_-*C*H_2_-(CH_2_)_12_-CH_2_-CH_3_), 29.9–29.1 (-CH_2_-CH_2_-(*C*H_2_)_12_-CH_2_-CH_3_), 27.8 (Al-CH_2_-CH(*C*H_3_)_2_), 25.8 (Al-CH_2_-*C*H(CH_3_)_2_), 22.8 (-CH_2_-CH_2_-(CH_2_)_12_-*C*H_2_-CH_3_), 20.5 (Al-*C*H_2_-CH(CH_3_)_2_), 14.0 (-CH_2_-CH_2_-(CH_2_)_12_-CH_2_-*C*H_3_) ppm.

**s-AlHAl**. ^1^H NMR (400 MHz, benzene-*d_6_*, 298 K): 7.04 (d, 4H, ^3^*J* = 8.8 Hz, aromatic *m*-CH), 6.82 (d, 4H, aromatic *o*-CH), 2.91 (bs, 1H, AlHAl), 2.48 (t, 4H, ^3^*J* = 7.8 Hz, -C*H_2_*-CH_2_-(CH_2_)_12_-CH_2_-CH_3_), 2.43 (s, 12H, N-CH_3_), 1.53 (m, 4H, -CH_2_-C*H_2_*-(CH_2_)_12_-CH_2_-CH_3_), 1.62 (m, 4H, Al-CH_2_-C*H*(CH_3_)_2_), 1.41–1.23 (m, 48H, -CH_2_-CH_2_-(C*H_2_*)_12_-CH_2_-CH_3_), 1.31 (m, 4H, -CH_2_-CH_2_-(CH_2_)_12_-C*H_2_*-CH_3_), 0.94 (t, 6H, ^3^*J* = 7.0 Hz, -CH_2_-CH_2_-(CH_2_)_12_-CH_2_-C*H_3_*), 0.92 (d, 12H, ^3^*J* = 6.6 Hz, Al-CH_2_-CH(C*H_3_*)_2_), 0.87 (d, 12H, ^3^*J* = 6.6 Hz, Al-CH_2_-CH(C*H_3_*)_2_), 0.10 (d, 8H, ^3^*J* = 7.0 Hz, Al-C*H*_2_-CH(CH_3_)_2_) ppm. ^13^C NMR (100 MHz, benzene-*d_6_*, 298 K): 143.8 (quaternary aromatic C), 142.1 (quaternary aromatic C-N), 130.0 (aromatic *m*-CH), 119.9 (aromatic *o*-CH), 46.8 (N-CH_3_), 34.8 (-*C*H_2_-CH_2_-(CH_2_)_12_-CH_2_-CH_3_), 31.2 (-CH_2_-*C*H_2_-(CH_2_)_12_-CH_2_-CH_3_), 29.9–29.1 (-CH_2_-CH_2_-(*C*H_2_)_12_-CH_2_-CH_3_), 27.7 (Al-CH_2_-CH(*C*H_3_)_2_), 25.5 (Al-CH_2_-*C*H(CH_3_)_2_), 22.7 (-CH_2_-CH_2_-(CH_2_)_12_-*C*H_2_-CH_3_), 21.9 (Al-*C*H_2_-CH(CH_3_)_2_), 14.0 (-CH_2_-CH_2_-(CH_2_)_12_-CH_2_-*C*H_3_) ppm.

### 2.3. Polymerization Experiments

Ethene/1-hexene copolymerization experiments were performed in a Freeslate Parallel Pressure Reactor setup with 48 reaction cells (PPR48), fully contained in a triple MBraun glovebox operating under nitrogen. The cells, each with a liquid working volume of 6.0 mL, featured an 800 rpm magnetically coupled stirring, and individual online reading/control of temperature, pressure, monomer uptake, and monomer uptake rate. Experiments were carried out according to established experimental protocols [27,39,48,49,50,51], under the following experimental conditions: ISOPAR-G solvent; *T*_p_ = 100 °C; *p*(C_2_H_4_) = 12 bar (175 psi; monomer fed on demand in a semi-batch fashion); 1-hexene initial concentration in reaction solution of 3.7 *v*/*v*% was used (see Section 3.2). Conversion of 1-hexene was kept below 15% to ensure a constant comonomer concentration. All experiments were performed at least in duplicate.

Prior to the execution of a polymerization library, the PPR modules undergo “bake-and-purge” cycles overnight (8 h at 90–140 °C with intermittent dry N_2_ flow), to remove any contaminants and leftovers from previous experiments. After cooling to glovebox temperature, the module stir tops are taken off, and the 48 cells are fitted with disposable 10-mL glass inserts (preweighed in a Bohdan Balance Automator, Mettler–Toledo, Milan, Italy) and polyether ether ketone (PEEK) stir paddles. The stir tops are then set back in place, and N_2_ in the reactors is replaced with ethene (ambient pressure).

The cells are then loaded with the appropriate amounts of a mixed alkane diluent (ISOPAR-G), containing TIBAL as a scavenger where appropriate, and 1-hexene. The system is then thermostated at the reaction temperature and brought to 12 bar of pressure with ethene. At this point, the catalyst injection sequence is started; aliquots of (a) an ISOPAR-G “chaser”, (b) a toluene solution of catalyst, (c) a toluene solution of the proper activator, and (d) a ISOPAR-G “buffer” are uploaded into the needle and subsequently injected into the cell of destination in reverse order, thus starting the reaction. This is left to proceed under stirring (800 rpm) at constant temperature and pressure with feed of ethene on demand until the desired monomer consumption has been reached (for reaction time; see Table S4), and quenched by overpressurizing the cell with 50 psi (3.4 bar) of dry air (preferred over other possible catalyst quenchers because in case of cell or quench line leakage oxygen is promptly detected by the dedicated glovebox sensor).

Once all cells have been quenched, the modules are cooled down to glovebox temperature and vented, the stir tops are removed, and the glass inserts containing the reaction phases are taken out and transferred to a centrifugal evaporator (RVC 2-33 CDplus, Martin Christ, Osterode am Harz, Germany), where all volatiles are removed, and the polymers are thoroughly dried overnight. The dry polymers were robotically weighed while still in the reaction vials, subtracting the prerecorded tare. Reaction yields are double-checked against online monomer conversion measurements. Polymer aliquots are then sent to the characterizations.

All polymers were characterized by means of high-temperature gel permeation chromatography (GPC) and ^13^C NMR spectroscopy. GPC curves were recorded with a Freeslate Rapid GPC setup, equipped with a set of two mixed-bed Agilent PLgel 10-μm columns and a Polymer Char IR4 detector. Calibration was performed with the universal method by using 10 monodisperse polystyrene samples (*M*_n_ between 1.3 and 3700 kDa).

Quantitative ^13^C NMR spectra were recorded by using a Bruker Avance III 400 spectrometer equipped with a high-temperature cryoprobe for 5-mm OD tubes, on 45-mg mL^−1^ polymer solutions in tetrachloroethane-*d*_2_ (with BHT added as a stabilizer, [BHT] = 0.4 mg mL^−1^). Acquisition conditions were: 45° pulse; acquisition time, 2.7 s; relaxation delay, 5.0 s; 2 K transients. Broadband proton decoupling was achieved with a modified WALTZ16 sequence (BI_WALTZ16_32 by Bruker, Billerica, MA, USA).

## 3. Results and Discussion

### 3.1. Synthesis and Characterization

**Scheme 1:**
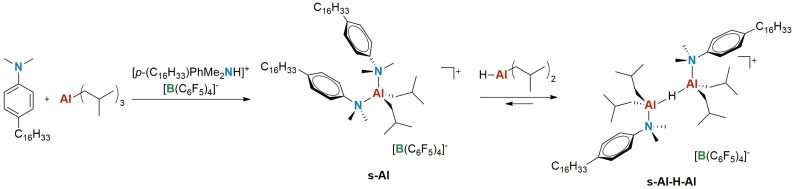
Synthesis of **s-Al** and **s-AlHAl**. DIBAL-H is shown as a monomer for simplicity.

**s-AlHAl** was synthesized by adapting the previously optimized procedure for **AlHAl** and its mononuclear precursor **Al** [27], replacing *N,N*-dimethylaniline (DMA) with its *p*-hexadecyl substituted analogue (DMA_C16_). Reaction of equimolar amounts of TIBAL, substituted aniline and the corresponding anilinium borate provided access to the mononuclear species **s-Al** [52], which was then converted into the dinuclear product by addition of one equivalent of di-*iso*-butylaluminum hydride (DIBAL-H; Scheme 1).

The alkyl substituent on the phenyl ring does not significantly affect the overall structure. The NMR spectra of **s-AlHAl** (see Supplementary Materials) are analogous to those of **AlHAl**, except for the additional set of signals of the *p*-hexadecyl residues. Likewise, the equilibrium constant (*K*_eq_) for the reaction

**s-Al** + [Al*i*Bu_2_H]_3_ ⇄ **s-AlHAl** + [Al*i*Bu_2_H]_2_ (1)

in toluene-*d*_8_ at 298 K was estimated to be approximately 24, corresponding to a Δ*G* ≈ −1.9 kcal/mol, implying that **s-AlHAl** is the largely dominant species in solution (Figure S3 in Supplementary Materials). These values are comparable within experimental uncertainty with those reported for **Al** and **AlHAl** under the same conditions (*K*_eq_ = 12; Δ*G* ≈ −1.5 kcal/mol) [27], indicating only marginal effects of the *para*-alkyl substituents on the relative stability of the mono- and dinuclear species. 

At the same time, solubility of **s-AlHAl** in saturated hydrocarbons turned out to be significantly enhanced. Pentane solutions with concentration of (at least) 7 × 10^−2^ M can be easily prepared. This value is well adequate, considering that typical catalyst concentrations in industrial reactors are as low as 10^−7^ M, and that (as noted before) less than 10^2^ equivalents of this novel activator are sufficient for efficient catalyst activation and impurity scavenging [27] (with MAO, which is the preferred cocatalyst in commercial processes, [Al]/[M] ratios between 10^3^ and 10^5^ are often necessary [9,53]). 

Importantly, the mononuclear species **s-Al** is far less soluble than **s-AlHAl**; this is convenient from the synthetic point of view because it allows for easy purification of the mononuclear species by washing with pentane (see Materials and Methods). The difference can likely be ascribed to the larger nature of the cation and the more delocalized charge of the dinuclear species [27], facilitating solvation by nonpolar solvents.

**Figure 2:**
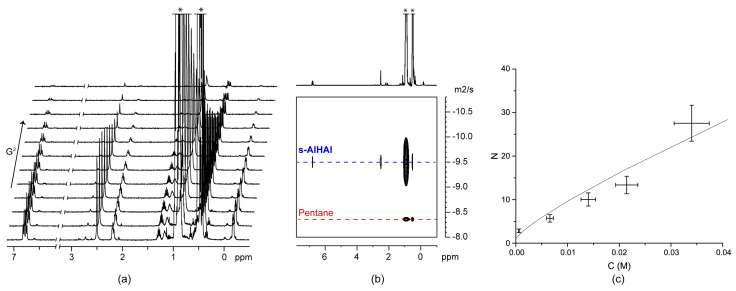
(**a**) Representative 1D diffusion NMR spectra of **s-AlHAl** at increasing gradient strength in pentane (with acetone-*d*_6_ coaxial capillary, 298 K, 33 mM) and (**b**) DOSY map derived therefrom; (**c**) trend of the average aggregation number (*N*) as a function of the analytical concentration of **s-AlHAl** derived from diffusion NMR measurements (trendline obtained by fitting experimental data based on the IK model; see ESI). *, solvent signals. A 15% and 10% error are assumed on aggregation number and concentration, respectively [43,44].

The high solubility of the alkyl-substituted cocatalyst also allowed recording NMR spectra in pure pentane at 298 K (Figure 2). Although the concentrations of **s-Al** and DIBAL-H could not be reliably estimated under these conditions, dinuclear **s-AlHAl** appears to be the dominant species in solution, similar to what is observed in toluene-*d*_8_. Furthermore, diffusion-ordered NMR spectroscopy (DOSY) experiments [54,55] could be carried out to investigate the tendency of **s-AlHAl** to aggregate in this representative low-polarity solvent [45].

The trend of average aggregation number (*N*) vs. concentration (*C*; Figure 2c) was estimated in the range 0.6–33 mM at 298 K. Experimental datapoints were fitted with the incremental *K_aggr_* (IK) model for indefinite self-association [43,44,46,56] to extrapolate the reference equilibrium constant *K*_aggr, IK_ = 3343 ± 1032 M^−1^ and the corresponding Δ*G*_aggr, IK_ = −4.8 ± 0.2 kcal/mol (see also Materials and Methods and Supplementary Materials). These values are comparable to those reported for the conceptually related zirconaziridine [Cp_2_Zr(*η*^2^-CH_2_N(C_18_H_37_)_2_)]^+^[B(C_6_F_5_)_4_]^−^ having long N-alkyl substituents (*K*_aggr_ = 15,589 ± 595 M^−1^; Δ*G*_aggr_ = −6.0 ± 0.1 kcal/mol in cyclohexane-*d*_12_, 298 K) [43], and are consistent with the formation of outer sphere ion pairs [4,43,57].

### 3.2. Polymerization Results

**Figure 3:**
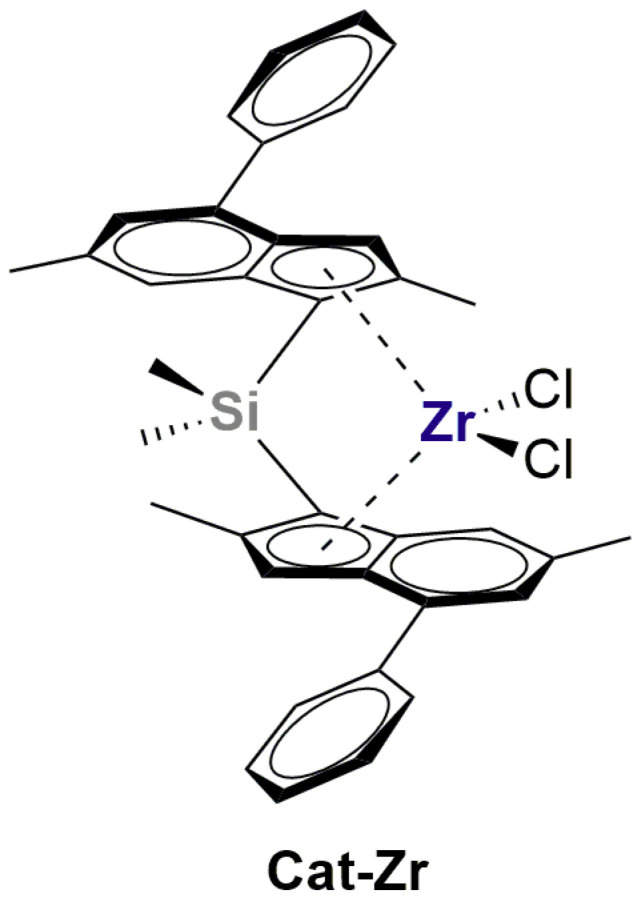
The *ansa*-zirconocene precatalyst **Cat-Zr** employed in polymerization screening.

The cocatalytic properties of **s-AlHAl** were tested in ethene/1-hexene copolymerization at 100 °C in a high-boiling alkane solvent by using *rac*-Me_2_Si(2,6-dimethyl-4-phenyl-1-indenyl)_2_ZrCl_2_ (Figure 3, **Cat-Zr**) [58] as a representative *ansa*-zirconocene dichloride precatalyst (see Materials and Methods and Supporting Information for further details). 

A well-established hydrocarbon-soluble activator, namely [HMeN(C_16_H_33_)_2_]^+^[B(C_6_F_5_)_4_]^−^ (RIBS), was used as a benchmark [36,37]. For catalytic activity RIBS alone is not sufficient because it can only serve as alkyl abstractor; therefore, TIBAL was added to the reaction system, thus acting as alkylating agent and scavenger [4,10]. **s-AlHAl** in principle does not require any additive [27], and it was therefore tested with and without added TIBAL. All polymerization experiments in the presence of TIBAL were performed by using [B]/[Zr] and [Al]/[Zr] ratios of 5 and 5.0 × 10^3^, respectively, which is fairly typical [26].

The results obtained under optimized conditions for each cocatalyst formulation are summarized in Table 1. The productivity obtained with **s-AlHAl**/TIBAL was appreciably higher than that with RIBS/TIBAL (Table 1, entries 1–2 vs. 3–5). Moreover, **s-AlHAl** performed well as a standalone cocatalyst, provided that its concentration was high enough to scavenge the reaction system. Optimum catalyst productivities without adding TIBAL were achieved at [**s-AlHAl**]/[Zr]~50 (Table 1, entries 6–8), instead of ~5 with TIBAL. In all cases, these Al levels are much lower than when using RIBS/TIBAL ([Al]/[Zr]~5 × 10^3^; Table 1, entries 6–8 vs. 1–2), and even more so compared with MAO (for which ([Al]/[Zr] up to 10^4^–10^5^ may be necessary for optimum performance [9,53]). No significant effects of activator nature and level on average copolymer molar mass and comonomer incorporation were observed.

## 4. Conclusions

In this paper, we introduced a novel molecular activator of the **AlHAl** family bearing long alkyl fragments on the aniline donor so as to enhance solubility in aliphatic hydrocarbons. This **s-AlHAl** species proved to be a competent cocatalyst in ethene/1-hexene copolymerizations in alkane diluent at high temperature. In combination with a representative *ansa*-zirconocene, catalyst productivities comparable to those with the better-known RIBS/TIBAL were obtained, with the advantage of working with a single-component activator at much lower [Al]/[Zr] ratios. These results represent a first demonstration that the **AlHAl** cocatalyst frame can be structurally amplified and tailored for specific applications; further variants are being prepared and screened in our laboratories, as will be reported in due course.

## Figures and Tables

**Table 1:** Ethene/1-hexene copolymerization results with precatalyst **Cat-Zr** in combination with different cocatalysts ^(a)^.

Entry	Activator	[B]/[Zr]	TIBAL	[Al_tot_]/[Zr]	$R_{p}$	$\bar{R_{p}}\;$	$M_{n}$	$\bar{M_{n}}\;$	$M_{w}$	$\bar{M_{w}}$	PDI	[H]
			µmol		kg μmol_Zr_^−1^ h^−1^	kg μmol_Zr_^−1^ h^−1^	kDa					mol%
1	RIBS	5	10	5.0 × 10^3^	0.19	0.22 (3)	57	56	141	139	2.5	7.4
2					0.25		56		137		2.5	7.2
3	s-AlHAl	5	10	5.0 × 10^3^	0.57	0.53 (5)	59	61	141	138	2.4	7.9
4					0.48		64		140		2.2	7.6
5					0.55		61		134		2.2	7.7
6		50	0	1.0 × 10^2^	0.42	0.50 (10)	59	55	134	134	2.3	7.9
7					0.48		51		134		2.6	7.9
8					0.61		54		134		2.5	8.0

^(a)^ in ISOPAR-G (*V*_tot_ = 6 mL), *p*_ethene_ = 12 bar, [H]_feed_ = 3.7 vol%, *T* = 100 °C, *n*_Zr_ = 2 nmol. *R*_p_, productivity; *M*_n_, number average molecular mass; *M*_w_, weight average molecular mass; PDI, polydispersity index; [H], 1-hexene incorporation into the polymer (determined by NMR spectroscopy).

## Data Availability

The data presented in this study are available in the Supplementary Materials.

## Supplementary Materials

The following supporting information can be downloaded at: https://www.mdpi.com/article/10.3390/polym15061378/s1, Figure S1: ^1^H NMR spectrum of [(p-C_16_H_33_)PhN(Me)_2_H]^+^[B(C_6_F_5_)_4_]^−^; Figure S2: ^1^H and ^13^C NMR spectra of **s-Al**; Figure S3: ^1^H and ^13^C NMR spectra of **s-AlHAl**; Figure S4: ^1^H NOESY spectrum of **s-AlHAl**; Figure S5: DOSY NMR maps of **s-AlHAl** in pentane at different concentrations; Figure S6: Ln(*I*/*I*_0_) vs. G^2^ plots relative to the DOSY maps in Figure S5; Table S1: DOSY NMR measured self-diffusion coefficient (*D*_t_), and estimated hydrodynamic radius (*r*_H_), hydrodynamic volume (*V*_H_) and average aggregation number (*N*) for TIBAL, **DMA_C16_** and **s-AlHAl** at different concentrations in pentane at 298 K; Determination of *K*_eq_.
