# Supplementary material for: A Hydrocarbon Soluble, Molecular and “Complete” Al-Cocatalyst for High Temperature Olefin Polymerization
Source: Polymers (Basel). 2023 Mar 9;15(6):1378. doi: 10.3390/polym15061378 (PMC10051415; doi:10.3390/polym15061378)
Supplement: Supplementary file 1 [file polymers-15-01378-s001.zip › polymers-2229170-supplementary.pdf]

Supporting Information

A Hydrocarbon Soluble, Molecular and "Complete" Al-Cocatalyst for High Temperature Olefin Polymerization

Ágnes Sallay [1,*], Imola Gecséné Tar [1], Zsuzsanna Mikházi [1], Katalin Takács [1], Cecilia Furlan [2] and Ulrike Krippner [2]

[1] Institute of Landscape Architecture, Urban Planning and Garden Art, Hungarian University of Agriculture and Life Sciences, 2100 Gödöllő, Hungary
[2] Institut für Landschaftsarchitektur, Universität für Bodenkultur Wien, 1180 Vienna, Austria
* Correspondence: sallay.agnes@uni-mate.com

Table of Content

1. Structural NMR spectroscopic characterization 2

2. Determination of K_{eq} 6

3. Diffusion NMR experiments 7

1. Structural NMR spectroscopic characterization

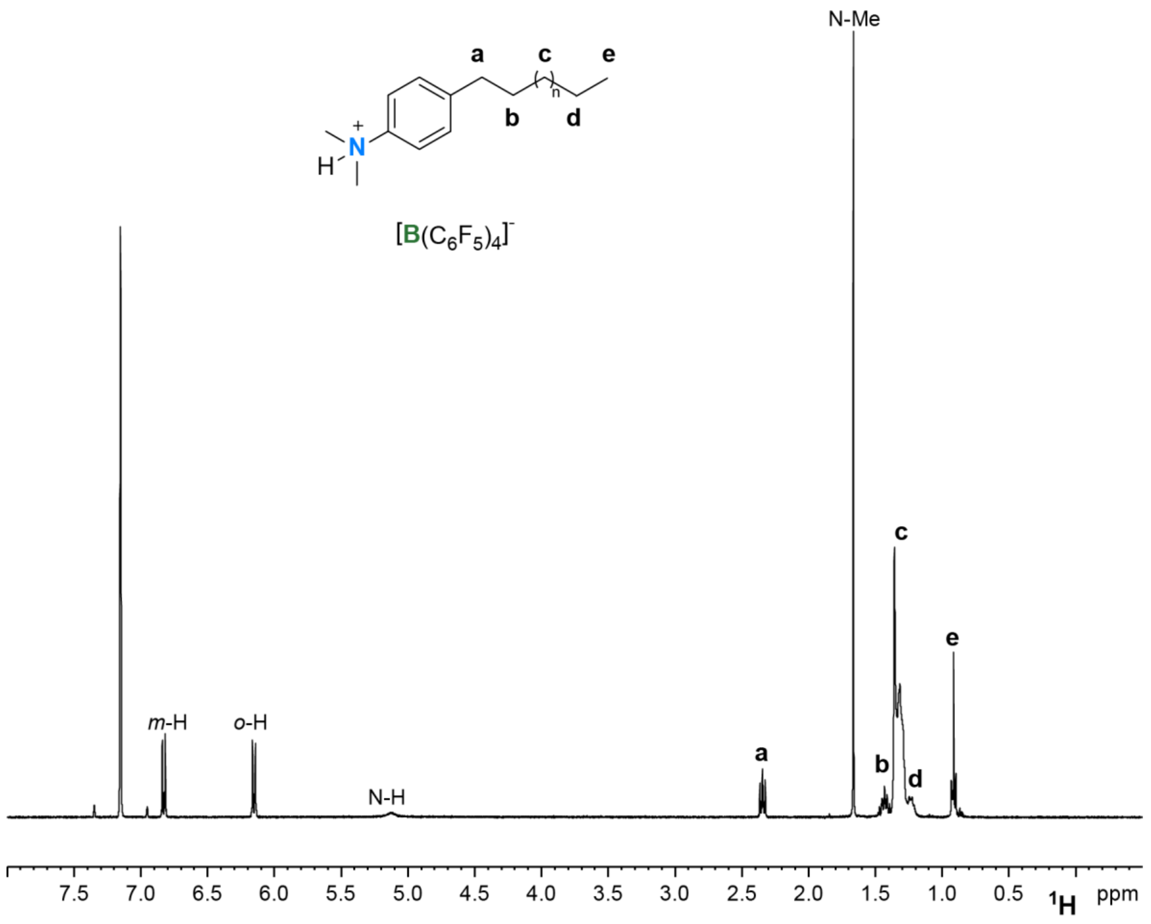

Figure S1. ^{1}H NMR spectrum (benzene-d_6, 298K) of $[(p\text{-}C_{16}H_{33})PhN(Me)_2H]^+[B(C_6F_5)_4]^-$.

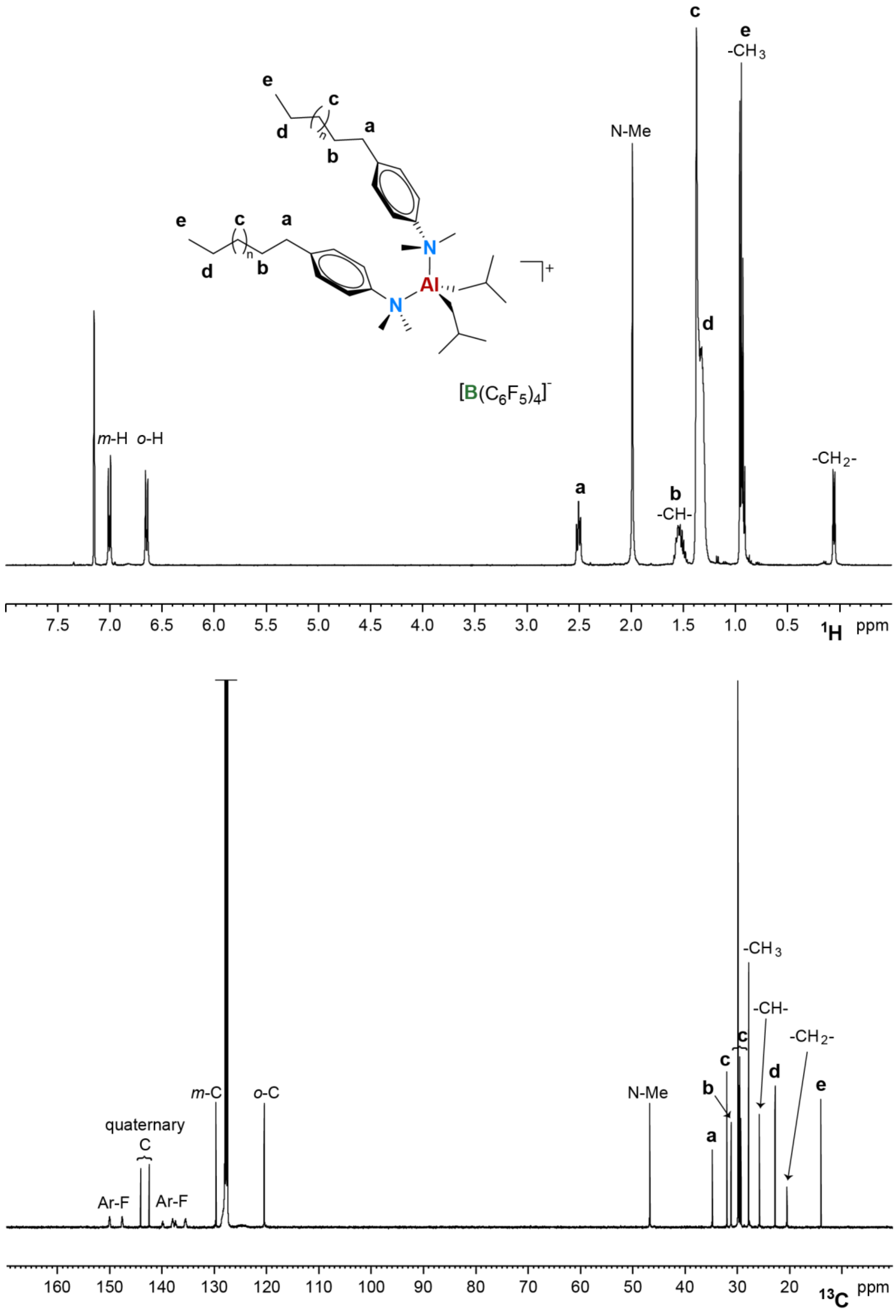

Figure S2. ^{1}H and ^{13}C NMR spectra (benzene-d_6, 298K) of **s-Al**.

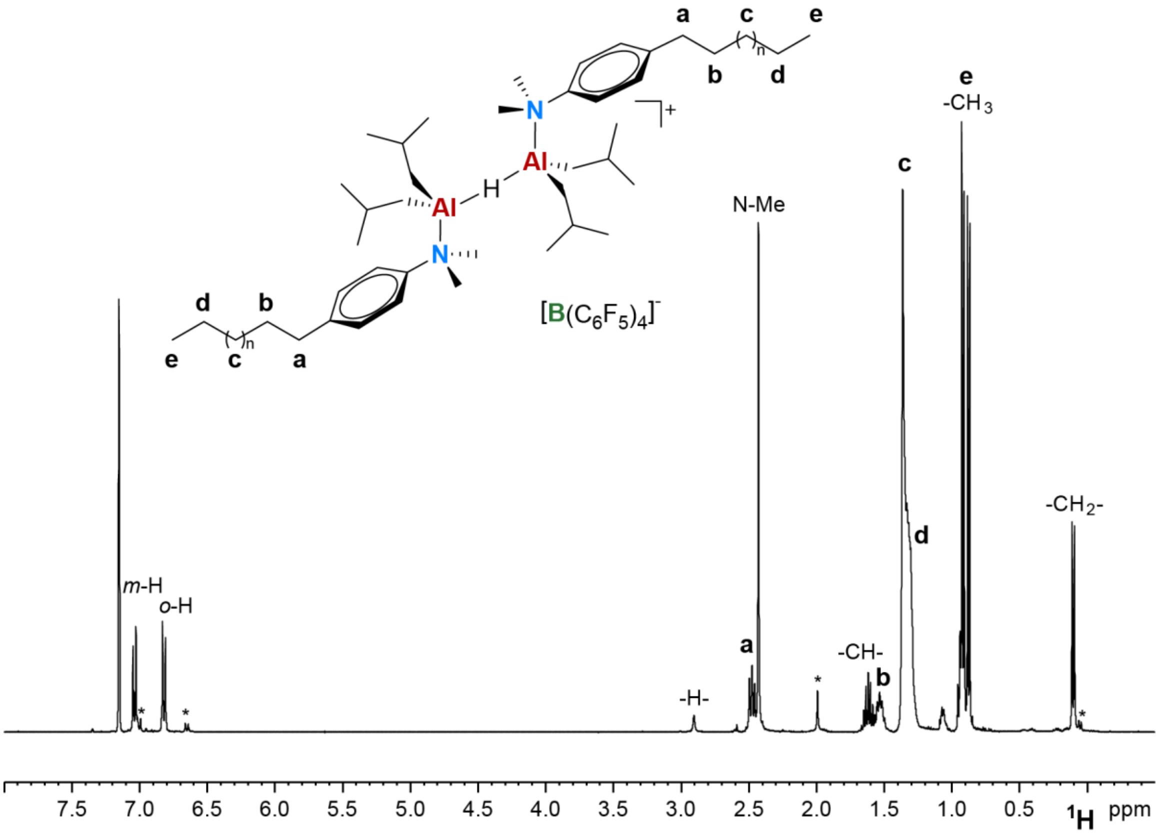

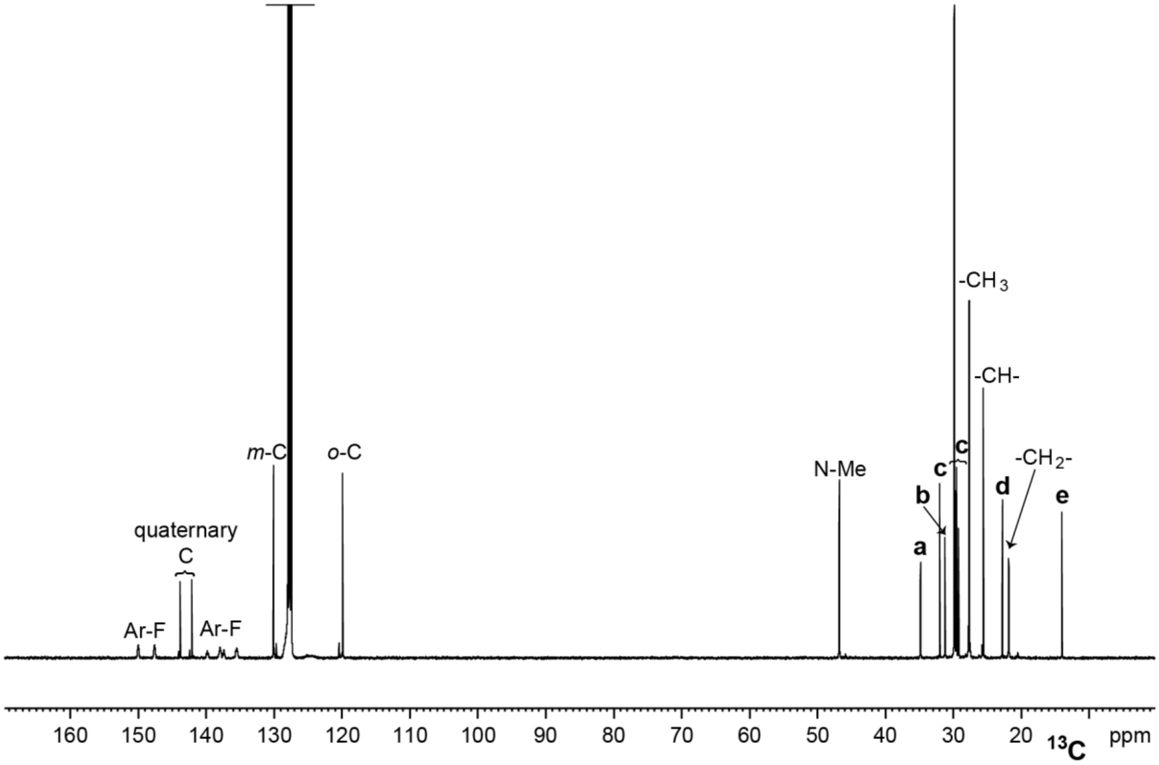

Figure S3. ^{1}H and ^{13}C NMR spectra (benzene-d_6, 298K) of **s-AlHAl**. *signals of residual **s-Al**.

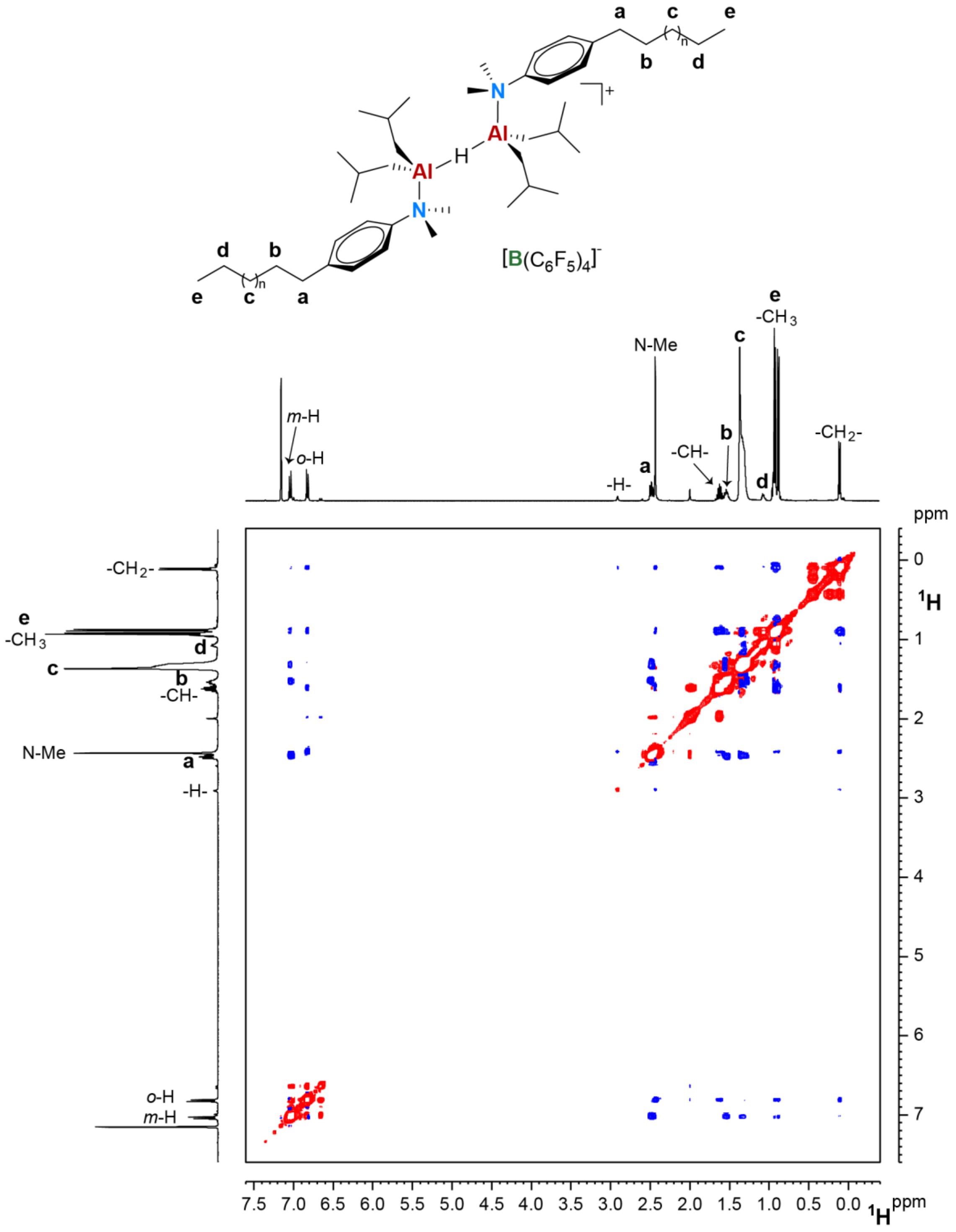

Figure S4. ^{1}H NOESY spectrum (benzene-d_6, 298K) of **s-AlHAl**.

2. Determination of K_{eq}

The K_{eq} for the equilibrium

$$\mathbf{Al}^{+} + (\mathrm{Al}i\mathrm{Bu_2H})_3 \rightleftarrows \mathbf{Al-H-Al}^{+} + (\mathrm{Al}i\mathrm{Bu_2H})_2$$

was estimated by measuring absolute concentrations of the Al-species by quantitative ^{1}H NMR spectroscopy in toluene-d_8 at 298 K, using an external standard:

$$K_{eq} = \frac{[Al-H-Al^{+}] \times [(AliBu_2H)_2]}{[Al^{+}] \times [(AliBu_2H)_3]} = \frac{21\,mM \times 0.37\,mM}{2.3\,mM \times 0.14\,mM} = 24$$

3. Diffusion NMR experiments

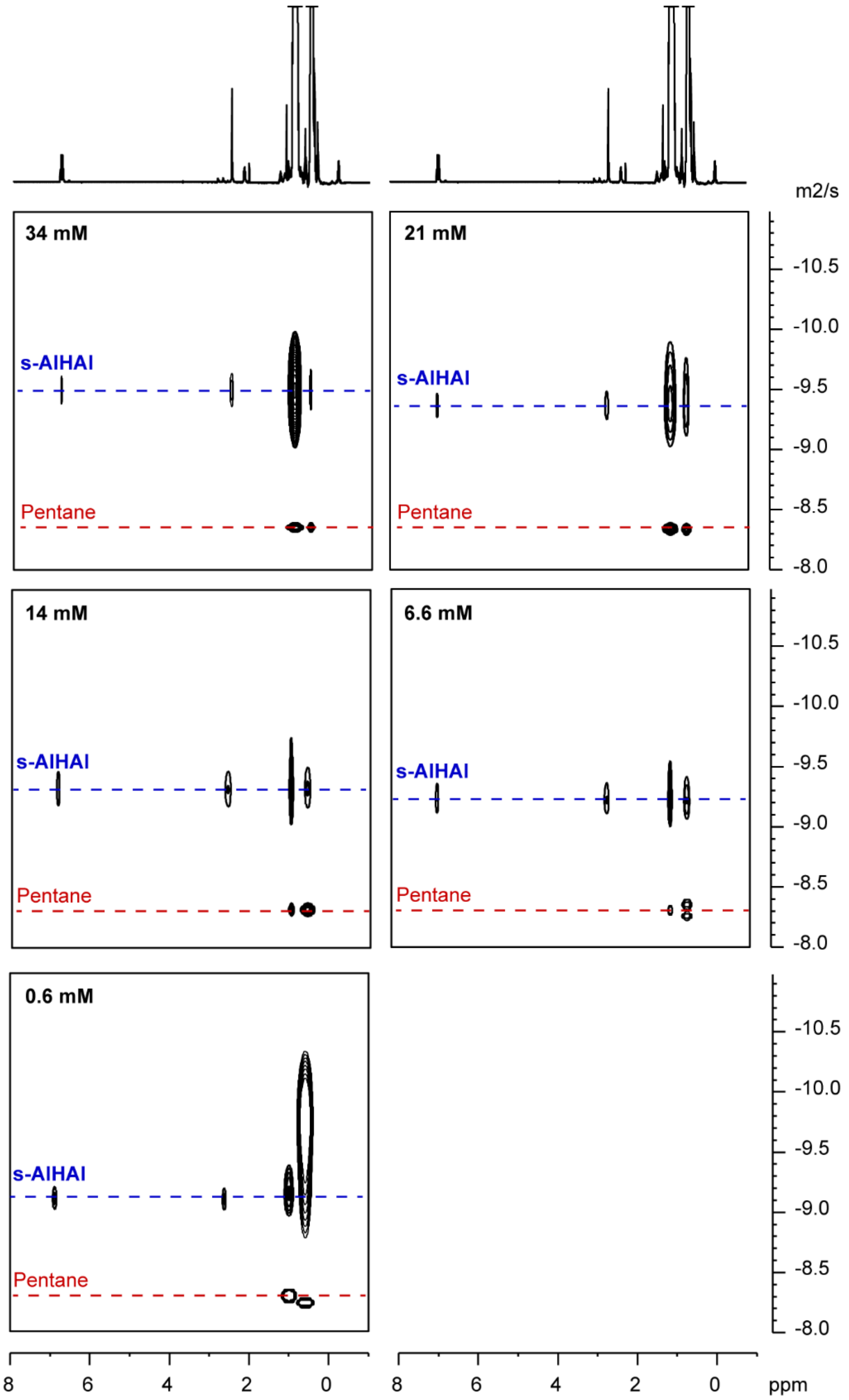

Figure S5. DOSY NMR maps of **s-AlHAl** in pentane at different concentrations (with acetone-d_6 coaxial capillary, 298 K).

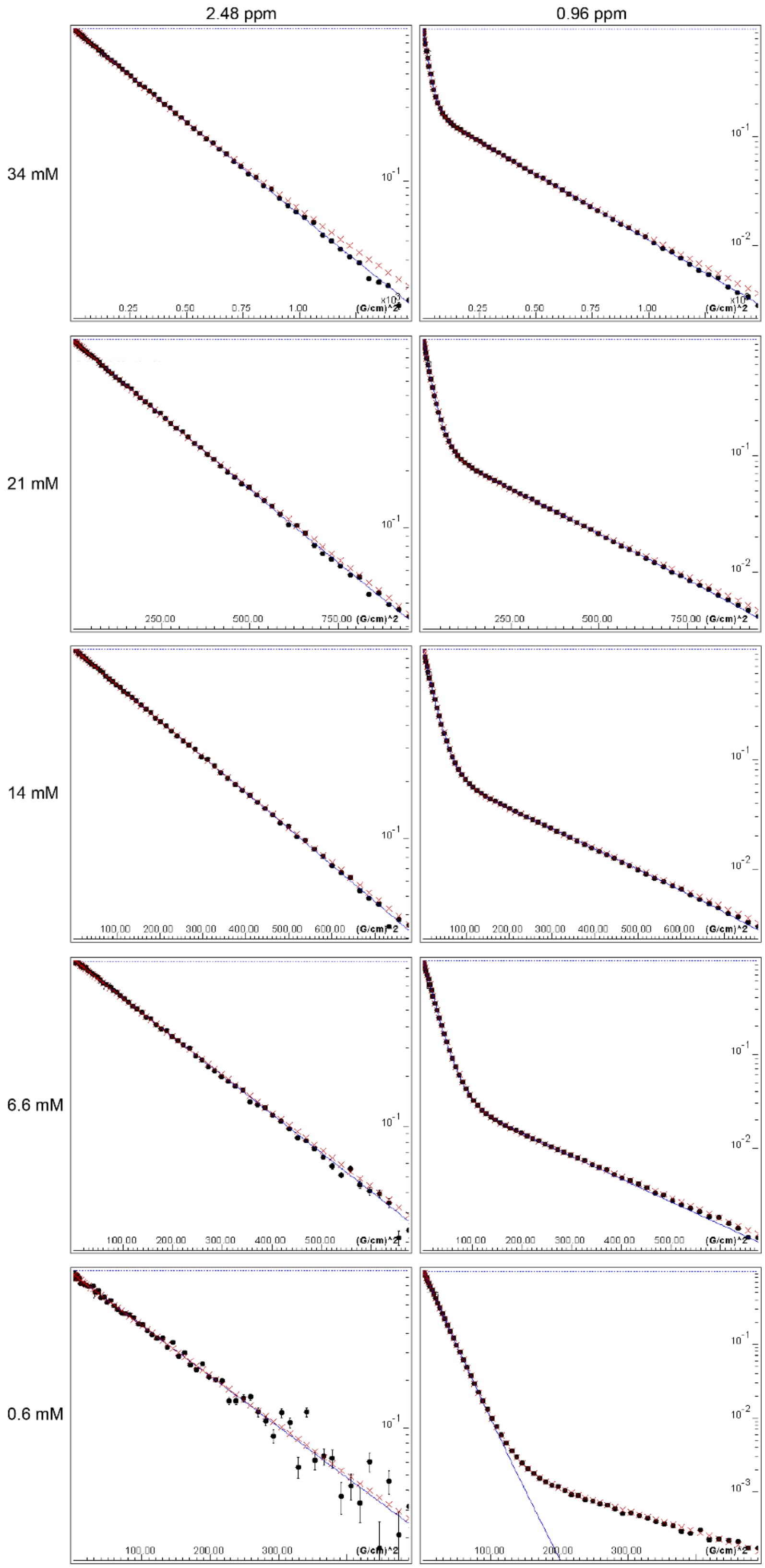

Figure S6. Ln(I/I_0) vs. G^2 plots relative to the DOSY maps in Figure S5, as obtained from the Bruker Dynamics Center software suite. Black dots = experimental points; red crosses = back-calculated points from ILT fitting; blue line = linear fitting (not considered here). The peak at 2.48 ppm corresponds to the N-Me group of **AlHAl**, the peak at 0.96 ppm to the overlapping $–CH_2–$ groups of **s-AlHAl** and pentane.

Table S1. DOSY NMR measured self-diffusion coefficient (D_t), and estimated hydrodynamic radius (r_H), hydrodynamic volume (V_H) and average aggregation number (N) for TIBAL, **DMA$_{C16}$** and **s-AlHAl** at different concentrations in pentane at 298 K.

Entry	Species	[s-AlHAl] (mM)	D_t (10^{10} m s^{-2})	r_H (Å)	V_H (Å^3)	N
1	TIBAL	-	28 ± 1	4.5 ± 0.2	381 ± 57	-
2	**DMA$_{C16}$**	-	22 ± 1	5.4 ± 0.3	659 ± 99	-
3	**s-AlHAl**	34	4.1 ± 0.2	25 ± 1	66999 ± 10049	27 ± 4
4	**s-AlHAl**	21	5.2 ± 0.3	20 ± 1	32498 ± 4874	13 ± 2
5	**s-AlHAl**	14	5.7 ± 0.3	18.0 ± 0.9	24416 ± 3662	10 ± 1
6	**s-AlHAl**	6.6	7.0 ± 0.4	14.9 ± 0.7	13849 ± 2077	5.7 ± 0.8
7	**s-AlHAl**	0.6	8.9 ± 0.5	11.8 ± 0.6	6878 ± 1031	2.8 ± 0.4
